# Non-targeted metabolomics data from solid fermented tobacco leaves derived from *Enterobacter hormaechei*

**DOI:** 10.1128/mra.00262-25

**Published:** 2025-11-13

**Authors:** Chen Liu, Zhen Wang, Jiandong Zhang, Lu Han, Jinbin Wei, Kai Song, Hongjing Yang, Zhipeng Zang, Yuzhen Gao, Liang Wang, Yaxin Zhang

**Affiliations:** 1 Gansu Tobacco Industry Co., Ltd, Lanzhou, China; 2 Enzyme Research and Development Department, Hzymes Biotechnology Co., Ltd., Wuhan, China; Rochester Institute of Technology, Rochester, New York, USA

**Keywords:** tobacco, *Enterobacter hormaechei*, non-targeted metabolomics, solid-state fermentation

## Abstract

The metabolomics of *Enterobacter hormaechei*-mediated solid-state fermentation in tobacco leaves is underexplored. Utilizing gas chromatography-mass spectrometry, we identified 300 dynamically regulated metabolites, including amino acids, carbohydrates, and organic acids. This dataset elucidates phase-specific biochemical shifts during fermentation, enhancing understanding of microbial-driven tobacco modification.

## ANNOUNCEMENT

Microbial fermentation under controlled temperature and humidity conditions facilitates the decomposition of macromolecules in tobacco leaves into small-molecule aromatic compounds. These include farnesyl acetone, damascenone, and megastigmatrienone derived from carotenoid degradation; benzyl alcohol, phenylethanol, and phenylacetaldehyde via phenylalanine conversion; and furfural/furfuryl alcohol generated through browning reactions (1, 2). Such compounds significantly enhance the aroma profile and sensory quality of tobacco products (3). Recent research indicates that selected microbial inoculants, including *Bacillus subtilis*, *Bacillus amyloliquefaciens*, *Saccharomyces cerevisiae*, and *Enterobacter hormaechei*, can enhance tobacco quality by modulating metabolic pathways (4, 5). For instance, *Bacillus altitudinis* inoculation increased total aroma production by 43% compared to natural fermentation (6). Similarly, *B. subtilis* ZIM 3 isolated from aged tobacco leaves exhibited robust enzymatic activity for starch and cellulose degradation (7).

Non-targeted metabolomics has emerged as a powerful tool for simultaneous detection of hundreds of endogenous metabolites in tobacco research (8, 9). This study integrates gas chromatography-mass spectrometry (GC-MS)-based metabolomics to systematically investigate the metabolic landscape of *E. hormaechei*-fermented tobacco leaves during solid-state fermentation.

Primary seed cultures began by inoculating −80°C glycerol stocks into Luria-Bertani (LB) medium (1:1,000 vol/vol) at 37°C for 8 h, followed by transferring 2 mL to fresh LB (1% vol/vol) for 16 h under the same conditions, after which cells were harvested, washed, and adjusted to 0.01 g/mL wet weight. Referring to the method described earlier (4), *E. hormaechei* suspensions (1% vol/wt, based on filler leaf dry mass) were aseptically sprayed onto tobacco leaves (30% moisture content). Control groups received equivalent sterile water. Treated leaves were sealed in bags and incubated at 30°C/70% humidity for 0, 6, 18, 24, and 72 h.

Samples (25 mg ± 10%) were weighed into 2 mL microcentrifuge tubes and mixed with 0.5 mL of pre-chilled (−20°C) acetonitrile:isopropanol:water (3:3:2, vol*/*vol*/*vol) and 3-4 zirconium beads (2 mm). Homogenization was performed using a high-throughput tissue grinder (30 Hz, 20 s pulses; 10 s intervals; eight cycles), followed by ice-water bath sonication (5 min). An additional 0.5 mL of chilled solvent was added, and samples were sonicated again (5 min) (10). After centrifugation (12,000 × *g*, 2 min), 500 µL of supernatant was transferred to new tubes, vacuum-concentrated to dryness (8–10 h), and derivatized with 80 µL methoxyamine hydrochloride (MEOX, 20 mg/mL; 60°C, 60 min). BSTFA-TMCS (99:1, 100 µL) was added, vortexed (30 s), and incubated (70°C, 90 min) (11). Post-centrifugation (14,000 × *g*, 3 min), 90–100 µL of supernatant was transferred to GC vials and analyzed by GC-time of flight within 24 h.

Separation was achieved using a DB-5MS capillary column (30 m × 250 µm, 0.25 µm film; Agilent J&W Scientific) with helium carrier gas (1 mL/min constant flow). Samples (1 µL) were injected in split mode (1:10 ratio) at 280°C. The temperature program initiated at 50°C (0.5 min), ramped to 320°C at 15°C/min, and held for 9 min. Transfer line and ion source temperatures were maintained at 320°C and 230°C, respectively. Full-scan mass spectra were acquired at 10 spectra/s with a *m/z* range of 67–1,000. Electron energy was set to −70 eV, with a 3 min solvent delay (12).

**Fig 1:**
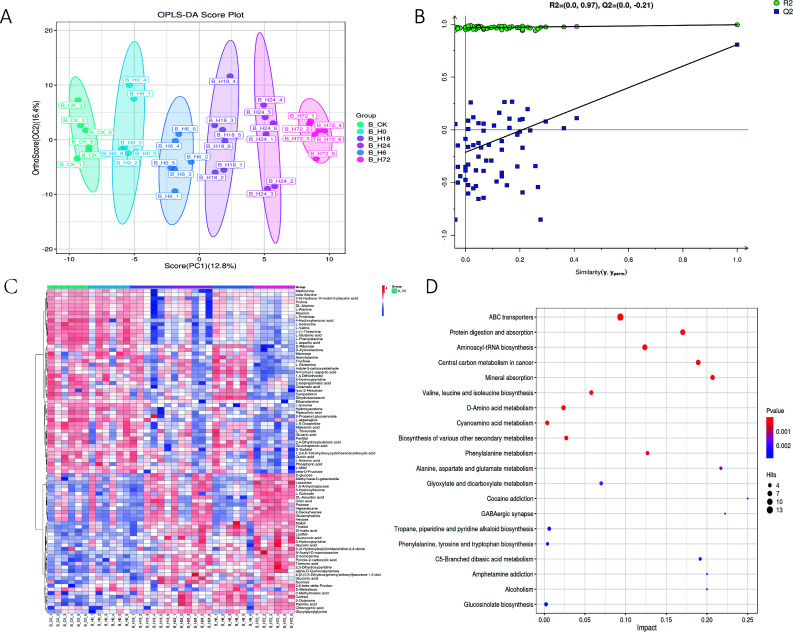
(**A**) Scatter plot of B_CK_vs_B_H0_vs_B_H6_vs_B_H18_vs_B_H24_vs_B_H72 OPLS-DA. (**B**) Displacement test plot of B_CK_vs_B_H0_vs_B_H6_vs_B_H18_vs_B_H24_vs_B_H72 OPLS-DA. (**C**) Heat map of up-/downregulated metabolites of different fermentation time and unfermented tobacco leaves. (**D**) KEGG map of different metabolites of fermented BCK vs BH24.

Metabolite identification was conducted using a GC-MS system coupled with a custom spectral library (Nuomi Metabolomics Biotechnology Co., Ltd., v3.1.5). Quantification and raw data preprocessing were performed with MS-DIAL v4.90 and XCMS v3.7.2 (13). The processed data matrix was imported into the SIMCA software (V14.1, Sartorius Stedim Data Analytics AB, Umea, Sweden) that utilized algorithms for normalization and standardization techniques, specifically the data matrix through mean-centering and unit variance scaling. Subsequently, orthogonal partial least squares-discriminant analysis (OPLS-DA) was performed, focusing on differential metabolites based on a *P* ≤ 0.05 and a variable importance for projection (VIP) score of ≥1 for metabolites associated with PC1 (14) (Fig. 1A and B) (Table 1). Finally, the MetaboAnalyst 5.0 and Kyoto Encyclopedia of Genes and Genomes (KEGG) databases were used to analyze the metabolic pathways of the identified differential metabolites (2). Data visualization was generated using Origin Pro 2023b (Origin Lab) and Python matplotlib v3.7.1 (15) (Fig. 1C and D).

**TABLE 1:** Differential metabolites based on VIP scores for PC1

Accession	VIP	Accession	VIP	Accession	VIP
1,3,-Tetrahydroxycyclohexanecarboxylic acid	1.72	D-Glucose	1.61	L-Threonate	1.10
1,4-Dithiothreitol	1.59	D-Homoserine	2.16	L-Valine	1.96
1,6-Anhydroglucose	1.68	DL-Alanine	1.27	L-Asparagine	1.76
2,3-Dihydroxypyridine	2.02	DL-Ascorbic acid	1.21	L-Aspartic acid	1.92
2,4-Dihydroxybutanoic acid	1.35	Dihydrolanosterol	1.65	L-Threonic acid	1.40
2,6-beta-delta-Fructan	1.01	Dl-malic acid	1.70	L-Tyrosine	1.18
2-(5-Hydroxy-1h-indol-3-yl)acetic acid	1.25	Ethanolamine	1.10	Lactitol	1.86
2-Deoxyhexose	1.14	Fructose	1.12	Lactulose	1.83
2-Hydroxypyridine	1.45	Glucoheptonic acid	1.53	Malbit	1.37
2-Isopropylmalic acid	1.13	Gluconic acid	1.15	Maleamic acid	1.71
2-Methylmaleic acid	1.23	Glucuronic acid	1.51	Mannose	1.21
2-Octanone	1.38	Glutamylvaline	1.54	Methionine	1.13
2-Propenyl glucosinolate	1.65	Glutaric acid	1.43	Methyl beta-D-galactoside	1.41
3-Hydroxyflavone	1.80	Glycolic acid	1.52	N-Acetyl-D-mannosamine	1.80
3-Hydroxypyridine	1.62	Glycylglycylglycine	1.29	N-Formyl-L-aspartic acid	1.44
4-Hydroxybenzoic acid	1.86	Heptadecane	1.48	Palmitic acid	1.82
4-[2-ethenyl]benzene-1,2-diol	1.08	Hexose	1.29	Pentitol	1.56
5-(4-Hydroxybutyl)imidazolidine-2,4-dione	1.31	Hydroxyacetone	1.28	Phosphoric acid	1.60
Abasine	1.41	Indole-3-carboxyaldehyde	1.33	Pipecolinic acid	1.48
Alanylalanine	1.04	L-(-)-Threonine	1.88	Proline	1.19
Campesterol	1.81	L-5-Oxoproline	1.72	Psicose	1.05
Chlorogenic acid	1.10	L-Alanine	1.29	Pyrrole-2-carboxylic acid	2.01
Citramalic acid	1.21	L-Glutamic acid	1.94	Quinic acid	1.43
Citric acid	1.00	L-Glutamine	1.43	Sucrose	1.35
Cortisol	1.13	L-Gulonate	1.11	Tartronic acid	1.81
D-Melezitose	1.20	L-Iditol	1.69	Threitol	1.49
D-Ribonate	1.42	L-Isoleucine	1.87	alpha-D-Quinovopyranose	1.65
D-Sorbitol	1.34	L-Phenylalanine	1.98	beta-Alanine	1.06
D-Xylonolactone	1.27	L-Prolimide	1.45	beta-D-Fructose	1.51
lyxo-2-Hexulose	1.37				

This study establishes the first multimodal metabolomics framework for *E. hormaechei*-fermented tobacco leaves, demonstrating GC-MS as a robust platform for monitoring fermentation-induced metabolic shifts. The data set serves as a valuable reference for optimizing microbial fermentation strategies in tobacco processing.

## Data Availability

The data sets underpinning the findings of this study have been deposited in the MetaboLights online repository under accession no. MTBLS12343.
